# Multi-Objective Optimization Using Deep Neural Network and Grey Relational Analysis for Optimal Lay-Up of CFRP Structure

**DOI:** 10.3390/ma18225104

**Published:** 2025-11-10

**Authors:** Min-Gi Kim, Jae-Chang Ryu, Chan-Joo Lee, Jin-Seok Jang, Do-Hoon Shin, Dae-Cheol Ko

**Affiliations:** 1 Department of Nanomechatronics Engineering, Pusan National University, Busan 46241, Republic of Korea; kkkkk@pusan.ac.kr; 2 Industrial Liaison Innovation Center, Pusan National University, Busan 46241, Republic of Korea; 3 Advanced Mobility Components Group, Korea Institute of Industrial Technology, Daegu 42994, Republic of Korea; cjlee80@kitech.re.kr (C.-J.L.);; 4 Aerostructure Business Department, Korea Air Lines, Busan 46712, Republic of Korea; dohshin@koreanair.com

**Keywords:** carbon fiber reinforcement plastic (CFRP), deep neural networks (DNN), pareto optimal solutions, gray relational analysis (GRA), multi-objective optimization

## Abstract

This paper proposes a multi-objective optimization method that integrates deep neural networks (DNN) with gray relational analysis (GRA) to optimize lay-up configurations for carbon fiber-reinforced plastic (CFRP) automotive components. Specifically, a lab-scale CFRP B-pillar structure was investigated to simultaneously maximize structural strength and failure safety. A DNN surrogate model was trained using finite element simulations of 2000 random stacking sequences to achieve high predictive accuracy. The trained model was then used to evaluate all possible lay-up combinations to derive Pareto optimal solutions. Gray relational analysis was subsequently employed to select the final optimal configurations based on designer preferences. The selected lay-up designs demonstrated improvements in both strength and failure safety. To validate the proposed framework, laboratory-scale CFRP B-pillar was fabricated using a prepreg compression molding process and subjected to bending tests. The experimental results confirmed an error below 5% and failure trends consistent with the simulation results, thereby validating the reliability of the proposed method. The proposed DNN-GRA approach enables efficient multi-objective optimization with reduced computational effort and flexibility in reflecting different engineering priorities.

## 1. Introduction

The automotive industry has increasingly focused on improving vehicle safety and reducing environmental impacts through lightweight structural designs. Among the various lightweight materials, carbon fiber-reinforced plastics (CFRP) are suitable for addressing this problem because of their superior specific strength, stiffness, and excellent fatigue performance compared to conventional metallic materials. Therefore, CFRP has been widely applied in structural components, particularly in safety-critical parts such as the B-pillar of vehicle. However, the effective design of CFRP structures remains challenging, particularly when multiple performance objectives, such as strength and crashworthiness, must be simultaneously optimized.

The growing demand for CO_2_ reduction has accelerated the development of hybrid and electric vehicles utilizing lightweight components [1]. In particular, extensive research has been conducted to replace traditional steel components with CFRP owing to its high strength and stiffness. Liu et al. developed a lightweight CFRP frame for electric vehicles [2], whereas Wang et al. applied CFRP to the design of automotive wheel rims to achieve a significant weight reduction compared with conventional materials [3]. The mechanical performance of CFRP structures is highly influenced by design variables, such as thickness [4], stacking angle [5], and lay-up sequence [6]. Accordingly, various optimization strategies have been applied to derive the optimal lay-up configurations. Owing to the nonlinear and complex relationship between design variables and mechanical properties, advanced surrogate modeling techniques have been increasingly utilized. Lee et al. performed thickness and lay-up optimization of CFRP-reinforced B-pillars using a genetic algorithm (GA) to maximize the bending stiffness [7]. Similarly, Shrivastava et al. optimized the stacking sequence of a double-double (DD) laminate for aerospace wing applications using an artificial intelligence (AI)-enhanced GA approach [8]. Moreover, Serban et al. applied various machine learning algorithms to predict fracture behavior based on CFRP lay-up configurations [9].

Engineering design problems typically involve multiple objectives and constraints that often conflict. For example, when the stacking sequence of a CFRP component is optimized with respect to strength alone, the fracture resistance can be inadvertently compromised. Thus, a multi-objective optimization approach is essential to simultaneously enhance multiple performance criteria. Recently, the Taguchi method combined with gray relational analysis (GRA) has been widely used for efficient multi-objective optimization with a reduced number of experiments. Shunmugesh et al. applied the Taguchi-GRA method to optimize microdrilling parameters for CFRP, confirming the influence of the material feed rate and spindle speed using an analysis of variance (ANOVA) [10]. However, the Taguchi method does not fully account for the interactions among the design variables, and its performance can vary depending on the selected experimental design, often leading to convergence toward local optima [11]. Sebaey et al. applied ant colony optimization (ACO) to obtain the optimal stacking sequences of CFRP laminates under low-velocity impact conditions [12]. Their study demonstrated a 21% reduction in the damaged area and an 8% improvement in the delamination threshold load compared with the base configuration. Although ACO shows strong performance in solving discrete optimization problems and offers stable results, it suffers from relatively slow convergence rates and lower solution precision [13]. Multi-objective genetic algorithms (MOGA) extend the capabilities of GA by applying Pareto optimality concepts to multi-objective problems. Beylergil et al. employed the MOGA to optimize the stacking sequence of CFRP plates subjected to eccentric loading [14]. The optimized configurations achieved improvements of 7% and 12% in strength and safety factors, respectively, compared with the base design. Although the MOGA effectively finds high-quality solutions in discrete spaces, it is computationally expensive and requires considerable time for convergence [15].

In addition to structural optimization, the DNN–GRA framework has been increasingly utilized in manufacturing process design and industrial monitoring applications, demonstrating its wide applicability in engineering practice. For instance, DNN–GRA-based models have been applied to optimize key component manufacturing parameters, industrial temperature measurement, and process control in high-temperature environments, bridging the gap between artificial intelligence and practical industrial systems [16]. These developments highlight the potential of AI-assisted optimization techniques to enhance process efficiency, reliability, and intelligent decision-making across multiple industrial domains.

Therefore, a methodology is required to account for the interactions among global design variables, such as deep neural networks (DNNs), to minimize computational time while maintaining optimization quality. The integration of Pareto optimality-based algorithms further enables the derivation of high-quality multi-objective solutions. In this study, a multi-objective optimization approach combining a DNN surrogate model with GRA is proposed to efficiently derive high-quality Pareto optimal solutions with reduced computational effort. The proposed approach was applied to the multi-objective lay-up optimization of a lab-scale CFRP B-pillar to simultaneously enhance strength and failure safety. Finally, to validate the proposed DNN-GRA optimization approach, a lab-scale CFRP B-pillar was fabricated using a prepreg compression molding (PCM) process, and the bending test was performed under the same conditions as the finite element analysis (FEA).

## 2. Method of Multi-Objective Optimization

**Figure 1:**
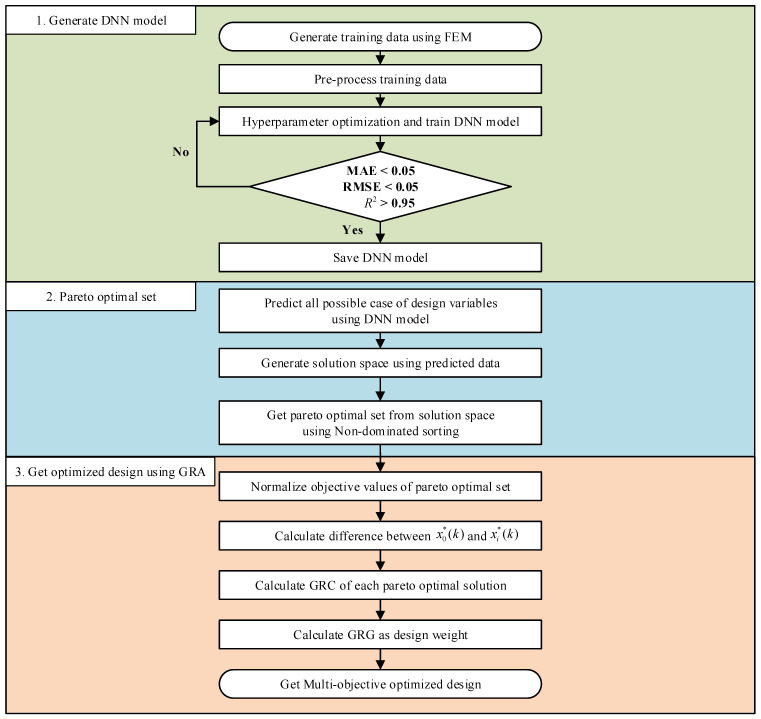
Procedure for multi-objective optimization combining DNN with GRA.

The procedure for the proposed multi-objective optimization methodology for the CFRP lay-up design, which integrates the DNN surrogate model and GRA, is illustrated in Figure 1. The overall process consists of three main stages: (a) construction of a DNN surrogate model based on training data through FEA, (b) generation of the solution space and extraction of the Pareto optimal set, and (c) selection of the optimal solution using GRA, according to the designer’s preferences.

First, to train the DNN surrogate model, a set of simulation data is generated using finite element analysis (Generate training data using FEA). The number of samples was determined based on the complexities and dimensions of the design variables. The FEA results were then normalized to a range between zero and one to enhance the learning performance of the DNN (Preprocessing the training data). Hyperparameter tuning was performed to determine the optimal DNN architecture (Hyperparameter optimization and training of the DNN model). The model with the best predictive performance on the validation set was saved for subsequent analysis (Save DNN model).

Second, the trained DNN model is used to predict the values of the objective function for all possible combinations of design variables (All possible cases of design variables are predicted using the DNN model). A one-to-one mapping of the predicted values was employed to generate the solution space (Generate solution space using the predicted data). From this solution space, the Pareto optimal set is extracted using a non-dominated sorting approach (Obtain the Pareto optimal set from the solution space using non-dominated point sorting).

Finally, to identify the most suitable solution according to the designer’s preferences, the GRA was applied to the previously derived Pareto optimal set. This involves several steps: (a) normalization of each objective function based on whether it follows the larger-the-better, smaller-the-better, or nominal-the-best characteristics (Normalized objective values of the Pareto optimal set), (b) calculation of the absolute difference between the reference and normalized values to determine the gray relational coefficient (GRC), (c) computation of the GRC for each Pareto solution, and (d) derivation of the gray relational grade (GRG) by applying weight factors to each objective function. The solution with the highest GRG value is selected as the optimal solution.

### 2.1. DNN Surrogate Model

Deep neural networks (DNNs), a class of artificial neural networks (ANNs) with multiple hidden layers, are machine learning models characterized by high adaptability, self-organization, and self-learning capabilities. These models are particularly well-suited for approximating complex nonlinear relationships between inputs and outputs, even when the training data are limited or noisy [17]. DNNs operate using interconnected layers of perceptron, where the output of each node is computed using Equation (1) [18]:

(1) $$Y_{j}=f\left(\sum_{i=0}^{n}Y_{i}w_{ij}+b_{j}\right)$$

where *Y_j_* is the output of node *j*; *f* is the activation function; *n* is the number of nodes; *Y_i_* is the output of node *i*; *w_ij_* is the connection weight; and *b_j_* is the bias term of node *j*.

Traditional activation functions such as sigmoid and hyperbolic tangents are widely used in backpropagation learning processes. However, as the network depth increases, these functions often suffer from the gradient vanishing problem, where the gradients become too small for effective learning [19]. To overcome this problem, the rectified linear unit (ReLU) activation function shown in Equation (2) was adopted for deeper architectures [20].

(2) $$f=\left\{\begin{array}{c} (x<0)f(x)=0\\ (x\geq 0)f(x)=x \end{array}\right.$$

**Figure 2:**
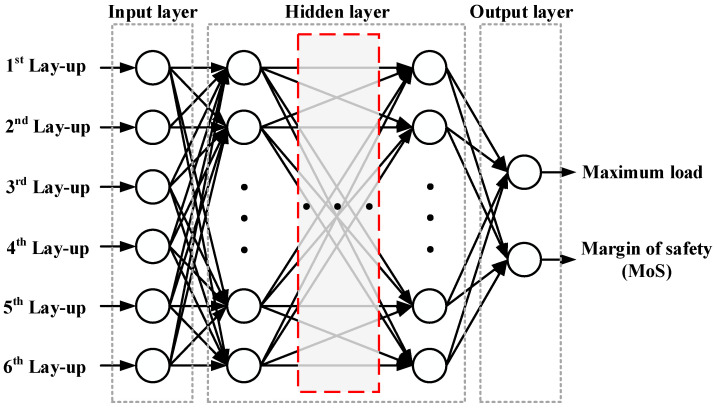
Schematic architecture of the deep neural network.

The ReLU function outputs zero for negative inputs and a linear response for positive inputs, thereby accelerating the training process and alleviating the vanishing gradient problem. In this study, the DNN was trained using an adaptive moment estimation (Adam) optimizer, which has demonstrated robust performance in navigating parameter spaces and has been widely adopted for DNN training tasks [21]. A schematic of the constructed deep neural network architecture is shown in Figure 2. The model comprises an input layer, several hidden layers, and two output nodes corresponding to the maximum load and margin of safety (MoS). ReLU activation was applied to each hidden layer, and the Adam optimizer was used for model training. Early stopping was implemented to prevent overfitting during the training. To assess the prediction accuracy of the trained model, three standard evaluation criteria were used: the Mean Absolute Error (MAE), Root Mean Square Error (RMSE), and coefficient of determination (*R*^2^). The definitions of these metrics are

(3) $$\mathrm{MAE}=\frac{1}{n}\sum \left|\hat{y}-y\right|$$

(4) $$\mathrm{RMSE}=\sqrt{\frac{1}{n}\sum {\left(\hat{y}-y\right)}^{2}}$$

(5) $$R^{2}=1-\left(\frac{\sum {\left(y-\hat{y}\right)}^{2}}{\sum {\left(y-\bar{y}\right)}^{2}}\right)$$

where *n* is the number of input data, $\hat{y}$ is the predicted value, *y* is the actual value, and $\bar{y}$ is the mean of the actual values. MAE quantifies the average magnitude of prediction errors and is less sensitive to outliers. RMSE, which squares the residual before averaging, penalizes larger errors more strongly, making it particularly sensitive to the impact of extreme deviations. *R*^2^ measures the proportion of variance in the dependent variable that can be predicted from the independent variables. Values of *R*^2^ closer to 1 indicate better model performance.

### 2.2. Pareto Optimal Set

Pareto optimality is a widely used concept in multi-objective optimization that acknowledges that the improvement of one objective function often leads to the degradation of another. In such scenarios, the goal is not to find a single global optimum but rather to identify a set of trade-off solutions that are non-dominated with respect to each other. This set is referred to as the Pareto optimal set [22].

**Figure 3:**
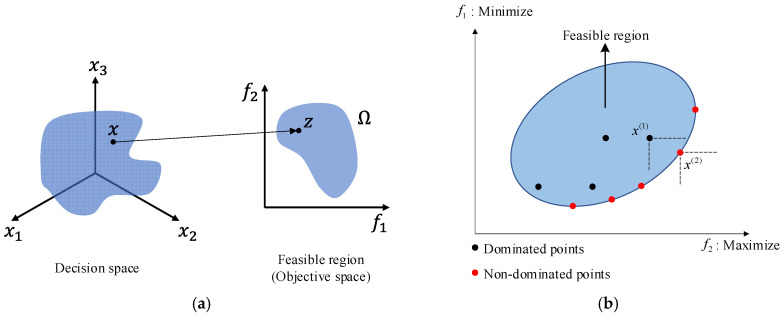
Schematic diagram of feasible region and non-dominated point: (**a**) Representation of decision space and feasible region; (**b**) Concept of dominance and non-dominance.

Mathematically, let Ω denote the feasible region in the objective space, and let *x* ∈ *X* represent a point in the decision space. The image of *x* under the objective function vector is denoted as *z* = *f*(*x*) ∈ Ω. The objective of Pareto optimization is to extract the set of non-dominated solutions from the feasible region defined by Equation (6) and illustrated in Figure 3:

(6) $$f\left(x\right)=\Omega ={\left(z_{1},z_{2},\cdots ,z_{M}\right)}^{T}$$

Here, a solution *x*^(1)^ is said to dominate another solution *x*^(2)^ denoted as *x*^(1)^ < *x*^(2)^, if the following two conditions are met [23]:

Condition 1 (Non-worseness):

(7) $$f_{j}\left(x^{\left(1\right)}\right)\leq f_{j}\left(x^{\left(2\right)}\right)\;\;for\;all\;j=1,2,\cdots ,M$$

Condition 2 (Strict improvement in at least one objective):

(8) $$f_{j}\left(x^{\left(1\right)}\right)<f_{j}\left(x^{\left(2\right)}\right)\;\;for\;at\;least\;one\;j\in \{1,2,\cdots ,M\}$$

**Figure 4:**
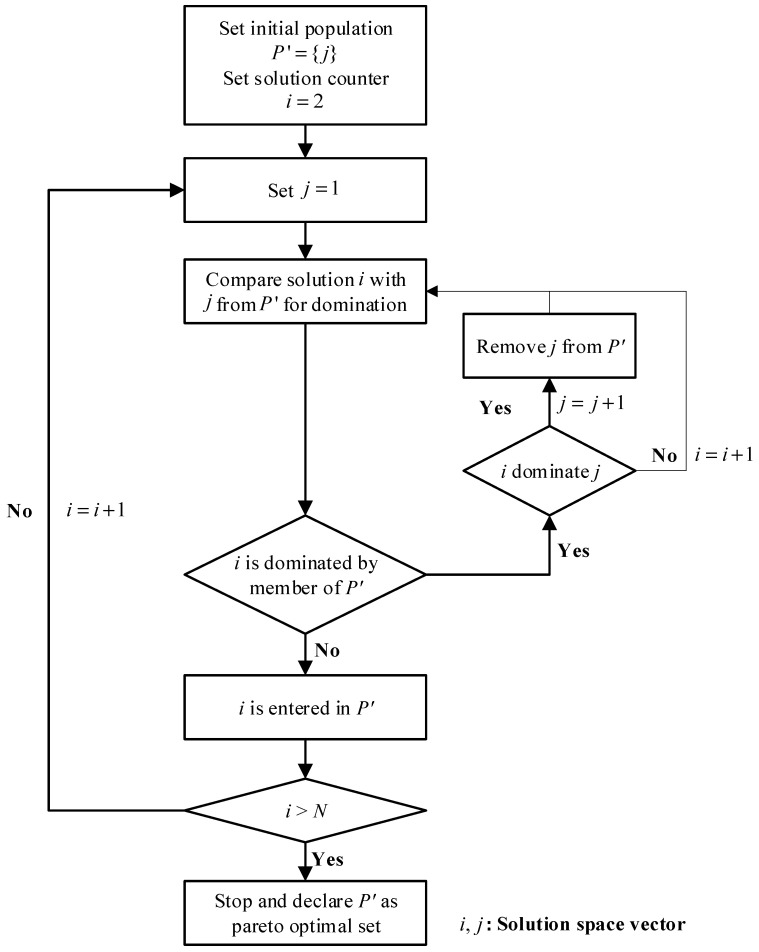
Flowchart of non-dominated point sorting.

A solution is considered non-dominated if no other solution in the feasible space satisfies both of these conditions. All non-dominated solutions collectively form the Pareto optimal set, which characterizes the boundary of the achievable trade-offs. The process of identifying the Pareto optimal set through non-dominated sorting is illustrated in Figure 4. In this process, the candidate solution *j* from the solution space is compared with every other solution *i*. If *i* dominates *j*, then *j* is removed from the candidate set *P*’. If *j* is not dominated by any solution *i*, it remains in *P*’. This iterative comparison is performed for all *i* ∈ {1, 2, …, *N*}, where *N* is the total number of feasible solutions. The final set *P*’ after completing all comparisons constitutes the Pareto optimal set.

The Pareto set obtained in this study represents a set of feasible CFRP lay-up configurations that simultaneously consider the trade-offs between the maximum structural strength and failure safety, which are considered as multi-objective functions. This set is used as the input for the subsequent decision-making phase using the GRA, where the most preferred solution is selected based on the designer’s specified weights.

### 2.3. Gray Relational Analysis

In this study, GRA was employed to select a single optimal solution from the Pareto optimal set, reflecting the designer’s specified weighting of the objectives. The decision-making process followed the structured four-step procedure detailed below [24]:

Step 1: Normalization of Pareto optimal values

The values predicted in the Pareto optimal solution were normalized based on the following objective characteristics: larger-the-better (LTB), smaller-the-better (STB), or nominal-the-best (NTB). The normalized value $x_{i}^{*}(k)$ for the *i*th solution and the *k*th objective function was calculated as:

(9) $$\mathrm{For}\;\mathrm{LTB}:\;x_{i}^{*}(k)=\frac{x_{i}(k)-\min_{k}x_{i}(k)}{\max_{k}x_{i}(k)-\min_{k}x_{i}(k)}$$

(10) $$\mathrm{For}\;\mathrm{STB}:\;x_{i}^{*}(k)=\frac{\max_{k}x_{i}(k)-x_{i}(k)}{\max_{k}x_{i}(k)-\min_{k}x_{i}(k)}$$

(11) $$\mathrm{For}\;\mathrm{NTB}:\;x_{i}^{*}(k)=\frac{\left|x_{i}(k)-T\right|-\min_{k}\left|x_{i}(k)-T\right|}{\max_{k}\left|x_{i}(k)-T\right|-\min_{k}\left|x_{i}(k)-T\right|}$$

where *x_i_*(*k*) is the original value of the *k*th objective for the *i*th solution, and *T* is the target value for the NTB objectives. In this study, both objectives followed the LTB characteristics.

Step 2: Calculation of absolute deviation from reference

To evaluate the proximity of each normalized value to the ideal value, the absolute deviation from the reference value (assumed to be 1) was calculated as follows:

(12) $$\Delta_{0i}(k)=\left|x_{0}^{*}(k)-x_{i}^{*}(k)\right|,\;\;\;k=1,2,\cdots ,n$$

where $x_{0}^{*}(k)=1$ is the reference (ideal) normalized value.

Step 3: Computation of gray relational coefficient (GRC)

The gray relational coefficient for each objective function was computed using:

(13) $$\mathrm{GRC}=\frac{\min_{i,k}\Delta_{0i}(k)+\xi \max_{i,k}\Delta_{0i}(k)}{\Delta_{0i}(k)+\xi \max_{i,k}\Delta_{0i}(k)}$$

where Δ_min_ and Δ_max_ are the minimum and maximum of all Δ_0*i*_(*k*), and ξ ∈ [0, 1] is the distinguishing coefficient (typically set to 0.5).

Step 4: Computation of gray relational grade (GRG)

The overall performance of each solution was evaluated by the gray relational grade $\gamma_{i}$, calculated as a weighted sum of the GRCs:

(14) $$\mathrm{GRG}=\Gamma_{0i}=\sum_{k=1}^{n}w_{k}\gamma_{0i}(k)$$

where *w_k_* denotes the weight assigned to the *k*th objective function. The weights reflect the designer priorities and satisfy the following constraints:

(15) $$\sum_{k=1}^{n}w_{k}=1$$

In this study, the proposed DNN-GRA methodology was applied to the multi-objective optimization of lay-up configurations for a lab-scale CFRP B-pillar. Two objective functions were defined to maximize the structural strength and failure safety under bending deformation. Both objectives were defined with a larger-the-better (LTB) characteristic, and GRA was performed using the corresponding normalization method. Specifically, normalized objective values were used to calculate the GRCs, which were then aggregated into a GRG through weighted summation. The solution with the highest GRG value was selected as the optimal final configuration. This approach enables an efficient optimal lay-up design that simultaneously satisfies both mechanical performance targets.

## 3. Results

### 3.1. Problem Definition and Design Variables

**Figure 5:**
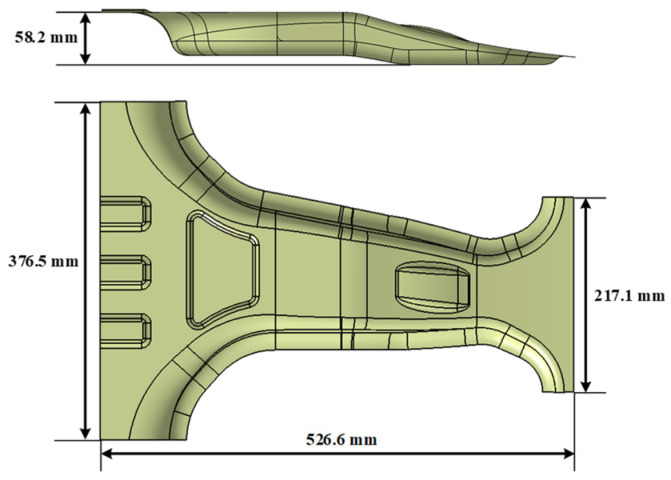
Three-dimensional model of Lab-scaled CFRP B-pillar.

The target structure for optimization in this study was a lab-scale CFRP B-pillar, which was modeled using CATIA V5 based on the geometry of typical automotive B-pillar components, as shown in Figure 5. For comparative evaluation with conventional steel body panels, a woven thermoset CFRP prepreg from TORAY was selected as the material for the lab-scale CFRP B-pillar. Six CFRP prepreg layers of 0.2 mm thickness per ply were stacked to achieve the same thickness as a DP590 steel panel of 1.2 mm thickness. The design variables used in this study were the fiber orientation angles of the six prepreg plies. Based on the feasible manufacturing and orthotropic behavior of the woven composites, the allowable ply angles were discretized in 15° increments as follows:

(16) $$\theta_{i}\in \left\{0{}^{\circ},15{}^{\circ},30{}^{\circ},45{}^{\circ},60{}^{\circ},75{}^{\circ}\right\}\;\;\;for\;i=1,2,\cdots ,6$$

The total number of combinations of design variables was 6^6^ (=46,656), representing all possible lay-up configurations within the specified angle set. Two objective functions were defined to maximize the structural strength and failure safety simultaneously. Specifically, two criteria were evaluated at a prescribed deformation level corresponding to a punch stroke of 14 mm: (a) maximum load (structural strength) and (b) maximum failure safety (MoS), which were calculated using the modified Tsai-Wu failure criterion. The Tsai-Wu failure criterion was employed to evaluate the failure behavior of each lay-up configuration under bending. This strength-based failure model considers the combined effects of in-plane stresses and provides a scalar failure index (FI), which is calculated as follows:

(17) $$F_{i}\bar{\sigma_{i}}+F_{ij}{\bar{\sigma }}_{i}{\bar{\sigma }}_{j}=1,\;\;\;i,j=1,2,6$$

Expanded into in-plane components, the equation becomes:

(18) $$F_{1}{\bar{\sigma }}_{1}+F_{2}{\bar{\sigma }}_{2}+F_{11}{\bar{\sigma }}_{1}^{2}+F_{22}{\bar{\sigma }}_{2}^{2}+F_{66}{\bar{\sigma }}_{6}^{2}+2F_{12}{\bar{\sigma }}_{1}{\bar{\sigma }}_{2}=1$$

The corresponding strength tensors, *F_i_* and *F_ij_* are defined using the tensile, compressive, and shear strengths of the material as follows:

(19) $$F_{1}=\frac{1}{X_{t}}-\frac{1}{X_{c}},\;\;\;F_{2}=\frac{1}{Y_{t}}-\frac{1}{Y_{c}},\;\;\;F_{11}=\frac{1}{X_{t}X_{c}},\;\;\;F_{22}=\frac{1}{Y_{t}Y_{c}},\;\;\;F_{66}=\frac{1}{S^{2}},\;\;\;F_{12}\approx -\frac{0.5}{\sqrt{X_{t}X_{c}Y_{t}Y_{c}}}$$

where *X_t_*, *X_c_*, *Y_t_*, and *Y_c_* are the tensile and compressive strengths in the fiber and transverse directions, respectively, and *S* is the in-plane shear strength. The material properties of the CFRP are listed in Table 1. Table 2 lists the strength values obtained from the technical datasheet of the manufacturer.

Although the Tsai–Wu index (FI = 1) identifies the onset of failure, it does not provide a quantitative measure of safety margin when FI < 1 [25,26]. To address this, a safety factor *R* was introduced as the ratio of the allowable stress to the actual stress, enabling a continuous measure of structural reliability [27]:

(20) $$R=\frac{\bar{\sigma }}{\sigma },\;\;\;\mathrm{viz}.,\;\;\;\left\{{\bar{\sigma }}_{1},{\bar{\sigma }}_{2},{\bar{\sigma }}_{6}\right\}=R\left\{\sigma_{1},\sigma_{2},\sigma_{6}\right\}$$

For the Tsai-Wu criterion, the safety factor *R* is determined by solving the quadratic relationship obtained from the polynomial form of the criterion:

(21) $$a\;R^{2}+b\;R-1=0$$

The analytical solution of this equation is:

(22) $$R=\frac{-b+\sqrt{b^{2}+4a}}{2a}$$

where the coefficients *a* and *b* are defined according to the Tsai-Wu strength tensor and the in-plane stress components as:

(23) $$a=F_{11}\sigma_{1}^{2}+F_{22}\sigma_{2}^{2}+F_{66}\sigma_{6}^{2}+2F_{12}\sigma_{1}\sigma_{2}$$

(24) $$b=F_{1}\sigma_{1}+F_{2}\sigma_{2}$$

Finally, the margin of safety (MoS) is defined as the surplus capacity beyond the applied load, given by:

(25) MoS=R−1

This metric offers intuitive insight: for instance, MoS = 1 indicates that the structure can withstand twice the applied load, while MoS = −0.5 implies failure unless the load is reduced by at least 50%. It should be noted that Equation (25) was derived under the plane stress assumption, and the Tsai-Wu coefficients (*F_i_*, *F_ij_*) were obtained based on an orthotropic material model for the CFRP lamina. The derivation steps presented above ensure the mathematical consistency between the general Tsai-Wu criterion and the scalar failure metric implemented in the FE simulation.

**Table 1:** Mechanical properties of CFRP laminate for structure analysis [28].

Property	Symbol	Value
Elastic Modulus in fiber direction (GPa)	$E_{11}$	65.01
Elastic Modulus in transverse direction (GPa)	$E_{22}$	65.01
Poisson’s ratio in 1–2	$\nu_{12}$	0.13
Shear Modulus in 1–2 (GPa)	$G_{12}$	12.69
Shear Modulus in 2–3 (GPa)	$G_{23}$	1.38
Shear Modulus in 1–3 (GPa)	$G_{13}$	1.38
Mass Density (g/cm^2^)		1.52

**Table 2:** Strength of CFRP.

Property	Symbol	Value
Tensile stress in fiber direction (MPa)	$X_{t}$	638
Compressive stress in fiber direction (MPa)	$X_{c}$	494
Tensile stress in transverse direction (MPa)	$Y_{t}$	633
Compressive stress in transverse direction (MPa)	$Y_{c}$	491
Shear strengh (MPa)	S	106

The deformation condition used in this study was based on the mechanical characteristics of automotive body panels with 1.2 mm-thick steel sheets, which generally exhibit elastic load responses up to 1 kN from the results of a previous study [7]. To provide a comparable reference to this benchmark while also enabling the evaluation of failure behavior in CFRP lay-ups, punch strokes of 12, 14, and 16 mm were initially examined. For each case, the margin of safety (MoS) and maximum load were evaluated for ten randomly selected lay-up configurations, as summarized in Table 3.

The results showed that the load of all selected configurations exceeded 1 kN at a punch stroke of 14 mm, and the MoS as a failure safety indicator showed meaningful differences with various distributions. By contrast, no failure was predicted at a stroke of 12 mm for any configuration, which was inadequate for the evaluation of failure-related characteristics. Furthermore, several lay-ups at a stroke of 12 mm produced loads below the 1 kN threshold, which restricted the evaluation of structural performance and made the condition unsuitable for comparative analysis. At a punch stroke of 16 mm, all lay-up configurations were predicted to fail, making it difficult to evaluate the trade-offs between the two objective functions, because every solution collapsed into a failure state. Therefore, a punch stroke of 14 mm was selected as the evaluation condition for lay-up optimization in this study because it represented a suitable distribution of values for strength assessment and damage progression.

### 3.2. Multi-Objective Optimization

**Figure 6:**
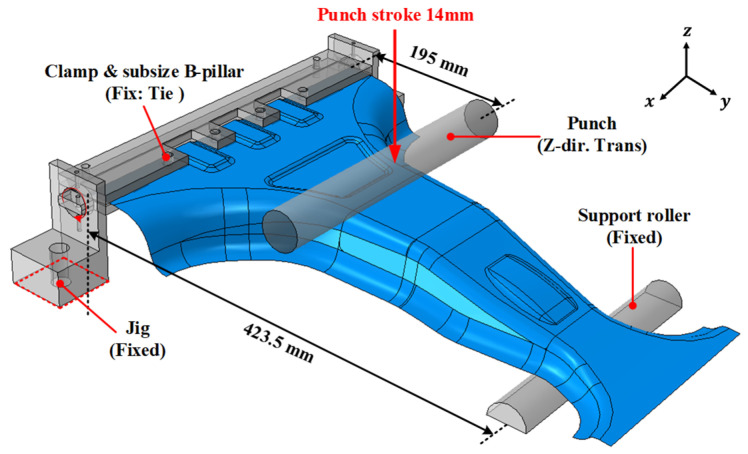
Boundary condition and impact position of lab-size CFRP B-pillar simulation.

**Figure 7:**
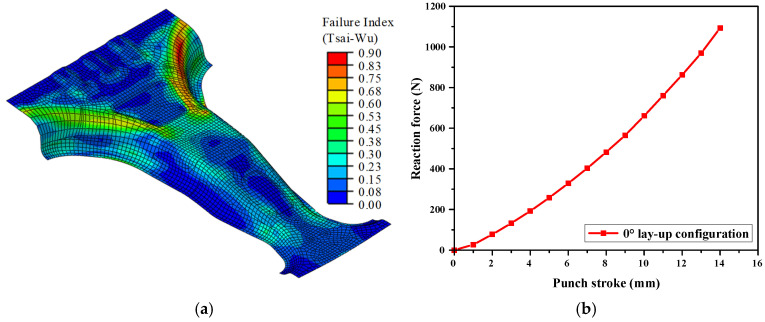
Results of FE simulation for 0° lay-up configuration of the lab-scaled CFRP B-pillar. (**a**) Failure index; (**b**) Stroke–force curve.

To construct the deep neural network (DNN) surrogate model, a finite element analysis (FEA) was conducted for 2000 randomly selected lay-up configurations to evaluate their structural strength and failure safety. The maximum load and MoS values for each lay-up configuration of the lab-scale CFRP B-pillar were obtained from the FEA under bending conditions, simulating a side impact, as illustrated in Figure 6. A linear static analysis of bending was adopted as an experimental and numerical surrogate to reproduce the local bending and compressive loads acting on the B-pillar during a side collision. This simplified bending model allows a controlled and repeatable evaluation of bending-dominated behavior while maintaining a direct correlation with the deformation mode of the full-scale component. The boundary conditions were defined to replicate the laboratory setup, with both supports fixed in the vertical direction and the punch displacement-controlled, ensuring consistency between the numerical and experimental results. The position of the applied load was based on the bumper height of the test vehicle [29,30]. The FEA model utilized 3D shell elements, and the two objective values, the maximum load and margin of safety (MoS), were normalized to the range of 0–1 using min-max normalization to improve the DNN learning performance. The representative FEA results for the 0° lay-up configuration, including the failure index distribution and stroke–force responses, are illustrated in Figure 7. This example demonstrate how the maximum load and MoS are extracted from the simulations and subsequently used as training data for the DNN surrogate model.

The dataset was divided into three subsets using random sampling: 70% for training, 15% for validation, and 15% for testing. The learning rate for the DNN model was set to 0.001, and the ReLU activation function was used in combination with the Adam optimizer. The maximum number of training epochs was set to 300 with early stopping to prevent overfitting when the validation loss plateaued. The final DNN model structure consisted of five hidden layers, each with 1000 nodes. This configuration was selected based on a systematic hyperparameter tuning process that evaluated various layer and node combinations to balance accuracy, generalization, and computational efficiency. Each candidate architecture was trained and validated using the same dataset, and its performance was quantitatively assessed through mean absolute error (MAE), root mean square error (RMSE), coefficient of determination (*R*^2^), and validation loss trends. Deeper networks tended to overfit the training data, while shallower ones exhibited insufficient nonlinear mapping capability and lower predictive accuracy. The selected architecture achieved stable convergence, minimized generalization error, and provided consistent predictions across multiple training iterations. The complete model is presented in Table 4. This architecture yielded excellent prediction accuracy, with MAE of 0.023, RMSE of 0.038, and R^2^ of 0.978 for the test set, as presented in Table 5.

**Figure 8:**
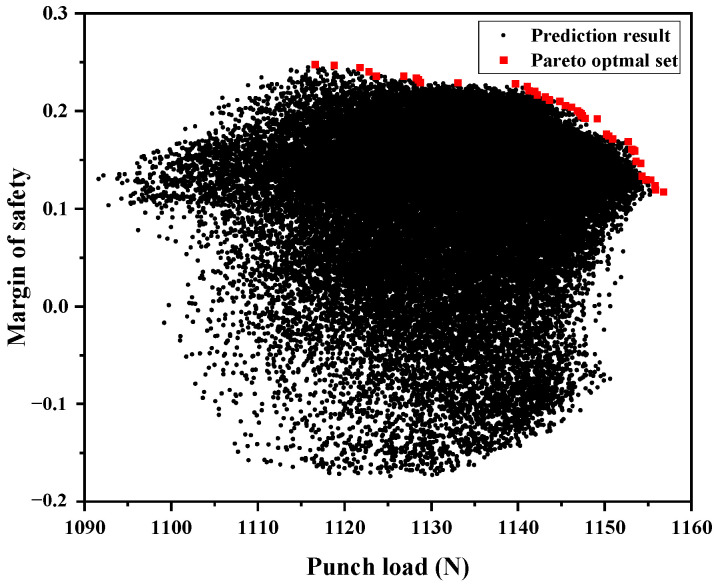
Solution space and pareto optimal set.

Using the trained DNN surrogate model, 46,656 possible lay-up configurations were evaluated to predict the load and MoS values. Subsequently, these predictions were used to generate the full solution space, as shown in Figure 8. From this space, 43 non-dominated solutions were identified using the Pareto sorting procedure described in Figure 4, and the lay-up configurations are listed in Table A1 in Appendix A.

To determine the optimal design considering the weight factor of the designer for the multi-objective function, which means that the designer can evaluate the multi-objective function as a single objective value according to its relative importance, gray relational analysis (GRA) was applied to the Pareto optimal set. For each Pareto optimal solution, the objective values were normalized using the larger-the-better characteristic, and the gray relational coefficient (GRC) was calculated using Equation (13), where a distinguishing coefficient of *ξ* = 0.5 was employed. The final gray relational grade (GRG) for each solution is then obtained from the summation of the GRCs with the assigned weights, as defined in Equation (14). Using this procedure, various weights can enable an optimal design reflecting the relative importance of the multi-objective function. Three different weights were applied to the two objective functions of structural strength and failure safety, where balanced design considered the same relative importance of structural strength and failure safety (*w*_Load_ = *w*_MoS_ = 0.5), load-focused design considered the relative importance of structural strength as 0.9 (*w*_Load_ = 0.9, *w*_MoS_ = 0.1), and MoS-focused design considered the relative importance of failure safety as 0.9 (*w*_MoS_ = 0.9, *w*_Load_ = 0.1), such as (a) GRG_1_ (Balanced design): *w*_Load_ = *w*_MoS_ = 0.5, (b) GRG_2_ (Load-focused design): *w*_Load_ = 0.9, *w*_MoS_ = 0.1, and (c) GRG_3_ (MoS-focused design): *w*_MoS_ = 0.9, *w*_Load_ = 0.1. Optimal lay-up configurations for each case were determined as follows: (a) GRG_1_ (Balanced): [0°/45°/60°/30°/0°/45°], (b) GRG_2_ (Load-focused): [45°/45°/0°/0°/30°/45°], and (c) GRG_3_ (MoS-focused): [0°/45°/60°/60°/60°/60°]. The GRG values corresponding to the determined lay-ups were 0.786, 0.950, and 0.950, respectively, as summarized in Table A1 in Appendix A. These values indicate that the selected lay-up configurations effectively achieved the optimized balance between structural strength and failure safety, which is consistent with the specified design intent.

**Figure 9:**
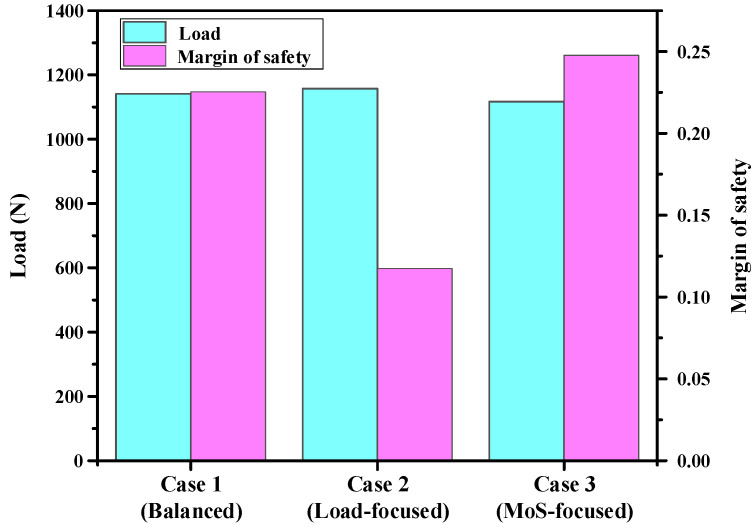
Performance comparison about each lay-up.

The GRG values of the three lay-ups predicted by the proposed DNN-GRA optimization framework were verified using FEA. The predicted results were compared with simulation results to assess the accuracy of the DNN model. All errors between the predicted and simulated results were found to be within 5%, demonstrating a high prediction accuracy, as shown in Table 6. The prediction error for the MoS was marginally higher than that for the maximum load, which can be attributed to the fact that the MoS was derived from the Tsai-Wu failure FI. Because the MoS involves a nonlinear combination of multiple stress components, small variations in the stress distribution can lead to amplified differences in the MoS value compared with the directly obtained load. A performance comparison of each case is shown in Figure 9. The load-focused design (Case 2) exhibited the highest strength. Although the maximum load increased slightly compared with the other cases, this design achieved the highest overall strength value. This result indicates that a slight difference in ply orientation can yield a significant improvement in the load capacity. In contrast, the MoS-focused design (Case 3) showed the lowest strength but provided the most significant increase in MoS. The balanced design (Case 1) indicated simultaneous and moderate improvements in both objectives. The effectiveness of the GRA optimization with different design intents was confirmed from the above three case studies. Finally, lab-scale CFRP B-pillars were manufactured using optimized lay-up configuration, and bending tests were performed to validate the reliability and feasibility of the proposed multi-objective optimization.

## 4. Experimental Verification

### 4.1. Manufacturing of Lab-Scaled CFRP B-Pillar

A lab-scale CFRP B-pillar was fabricated and tested to experimentally verify the performance of the optimal lay-up configurations using the proposed DNN-GRA method.

**Figure 10:**
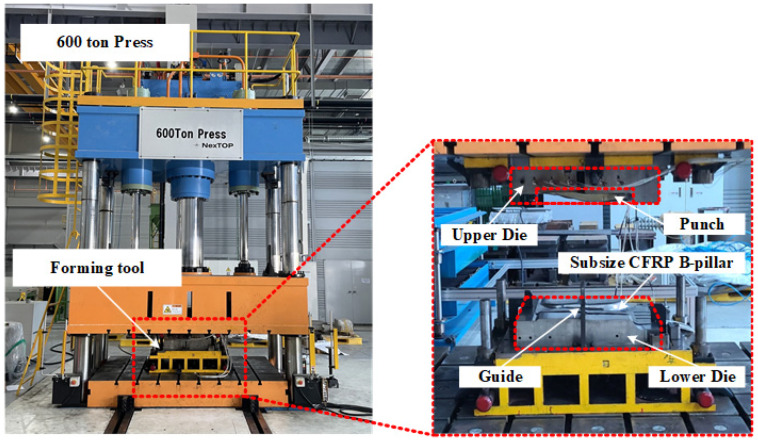
Experimental equipment for manufacturing of lab-size CFRP B-Pillar.

**Figure 11:**
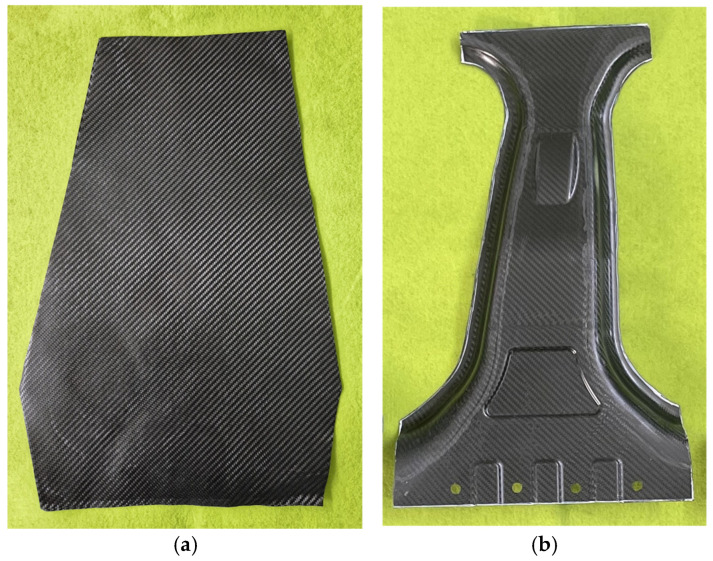
Experimental results of manufacturing lab-scale CFRP B-pillar through PCM process. (**a**) Before forming; (**b**) After forming.

Three lay-up configurations mentioned in Section 3.2 were used for the verification: Case 1 (balanced lay-up), Case 2 (load-focused lay-up), and Case 3 (MoS-focused lay-up). As shown in Figure 10, the lab-scale CFRP B-pillar was manufactured by prepreg compression molding (PCM), which consisted of an upper punch and a lower die. A hydraulic press with a capacity of 6000 kN was used to apply the required forming pressure. The tool temperature for forming and curing was maintained at 160 °C using embedded cartridge heaters. The twill-weave thermoset CFRP prepregs were stacked according to each lay-up case, as shown in Figure 11a, and placed into the heated tool. A high-temperature release agent was applied to the tool surface before preheating to prevent adhesion. After preheating for 1 min, compression forming was conducted under full press capacity. A dwell time of 3 min was used to complete the thermal curing process. After curing, the formed parts were demolded and air-cooled to room temperature. The fabricated lab-scale CFRP B-pillar is shown in Figure 11b and was used in subsequent bending tests to evaluate the structural performance and failure safety of the optimized lay-up configurations using the DNN-GRA method.

### 4.2. Bending Test

**Figure 12:**
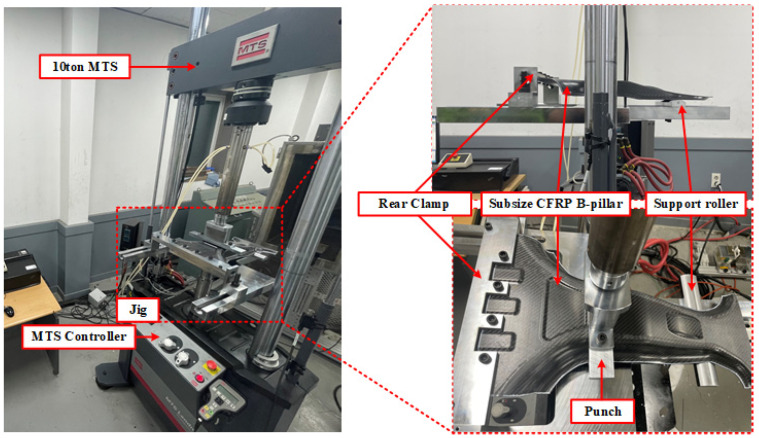
Experimental apparatus for bending experiment of Lab-size CFRP B-Pillar.

**Figure 13:**
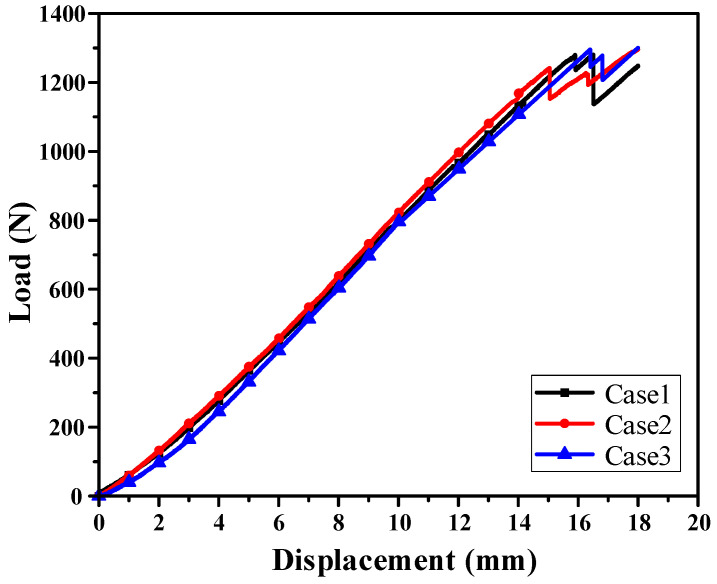
Results of bending experiment for each lay-up cases.

To validate the effectiveness of the proposed DNN-GRA method for multi-objective optimization, bending tests were conducted on a lab-scale CFRP B-pillar fabricated according to three optimal lay-up configurations. The tests were performed using a material testing system (MTS) of 100 kN capacity, where a specifically designed jig was used to test the lab-scale CFRP B-pillar, as shown in Figure 12. The test for each case was performed three times to ensure the reliability of the experimental results. The structural strength was evaluated by comparing the load resistance among all cases at a punch stroke of 14 mm, whereas the failure safety was evaluated from the load drop up to a punch stroke of 18 mm, because of the difficulty in directly quantifying the failure index from the bending tests. The average load–displacement curves obtained from three repeated experiments for each case are presented in Figure 13, and the corresponding experimental results are summarized in Table 7. The experimental results for a punch stroke of 14 mm showed good agreement with the FEM predictions, with errors of 1.08%, 0.64%, and 0.98% for Cases 1, 2, and 3, respectively. These small errors demonstrate the high reliability of the proposed DNN-GRA optimization approach. Case 2 exhibits the highest performance in terms of structural strength, followed by Cases 1 and 3. This result is consistent with the intended design priorities, thereby confirming the effectiveness of the proposed DNN-GRA optimization framework.

**Figure 14:**
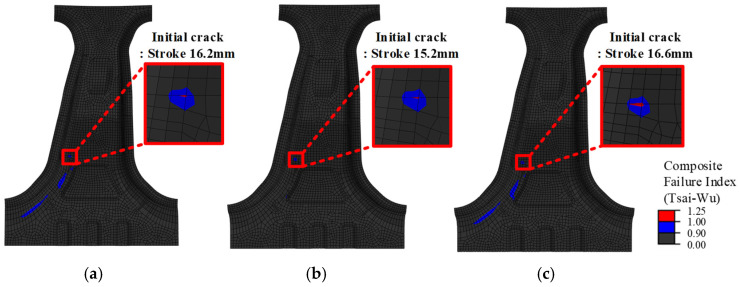
FEM results of initial crack locations for each lay-up case. (**a**) Case 1: Balanced; (**b**) Case 2: Load-focused; (**c**) Case 3: MoS-focused.

To evaluate the failure initiation, the FEM results at the initial damage points are shown in Figure 14. The punch strokes corresponding to the onset of cracking were 16.2 mm, 15.2 mm, and 16.6 mm for Case 1, Case 2, and Case 3, respectively. In the simulations, failure initiation was defined as the point at which the Tsai-Wu failure index (FI) reached or exceeded 1.0. The first occurrence of cracking in the experiment was observed at punch strokes of 15.91 mm, 15.05 mm, and 16.4 mm for Cases 1, 2, and 3, respectively. As summarized in Table 7, the deviation between the simulation and experimental results is less than 2%, confirming the predictive accuracy of the model. The failure in Case 1, which was designed with equal weighting of the two objective functions, was initiated between the punch strokes observed in Cases 2 and 3. Case 2, which was optimized for structural strength, failed 1.35 mm earlier than Case 3, corresponding to an 8.97% reduction, suggesting a greater susceptibility to damage.

**Figure 15:**
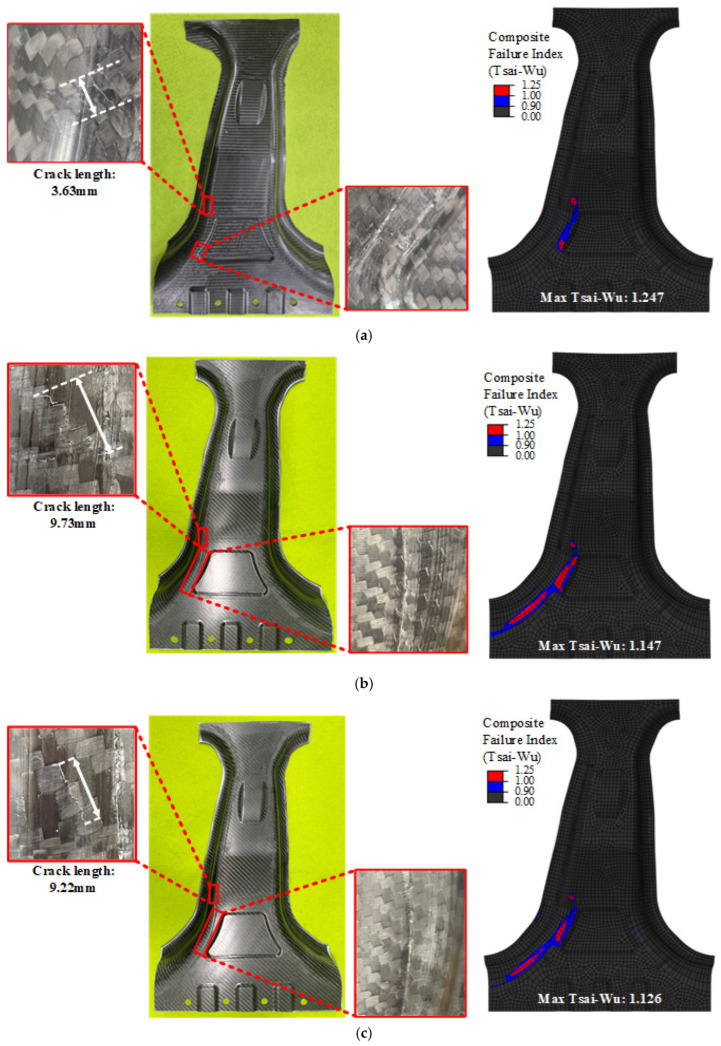
Comparison of Experiment and FEM result at punch stroke 18 mm. (**a**) case 1; (**b**) Case 2; (**c**) Case 3.

The crack propagation behavior at a punch stroke of 18 mm is shown in Figure 15. As illustrated in Figure 15a,c, Cases 1 and 3 exhibited similar crack locations and propagation patterns in both the simulation and experiments. However, Case 3, which was optimized to minimize the failure index, exhibited fewer cracks and lower predicted damage. The close agreement between the simulated crack locations and those observed experimentally further confirms the validity of employing the Tsai-Wu index as an effective criterion for damage initiation. In contrast, Figure 15b shows that Case 2 experienced a more concentrated crack zone. The Tsai-Wu failure index reached 1.247, which was the highest among all the cases, indicating stress concentration and early failure. This behavior is consistent with the early onset of cracking, as discussed above, confirming that Case 2 was the most vulnerable in terms of failure safety. These results clearly reveal the inherent trade-off between maximizing the structural strength (Case 2) and enhancing the failure safety (Case 3). The proposed DNN-GRA optimization framework explicitly captures this trade-off and provides a systematic basis for designers to select the most appropriate balance based on their priorities.

Overall, the bending tests showed a close agreement between the experimental observations and FEM predictions with respect to the maximum load, damage initiation, and failure propagation. These findings confirmed the validity of the proposed DNN-GRA optimization approach and demonstrated its effectiveness in generating high-performance lay-up configurations for multiple objectives.

## 5. Conclusions

In this study, a multi-objective optimization framework integrating a Deep Neural Network (DNN) and Gray Relational Analysis (GRA) is proposed to overcome the limitations of conventional methods in composite structure design. The framework was applied to optimize the lay-up configuration of a lab-scale CFRP B-pillar with the two objectives of maximizing structural strength and improving failure safety. Using FEM simulations and a DNN-based surrogate model, Pareto optimal solutions were identified, and three representative designs—balanced, load-focused, and MoS-focused—were selected using the GRA. The DNN model achieved high prediction accuracy, with deviations of less than 5% compared to the FEM results. Performance comparisons indicate that the load-focused design exhibits the highest structural strength, whereas the MoS-focused design exhibits superior failure safety. A balanced design offers moderate improvements in both objectives. Bending tests conducted under the same conditions as the simulations confirmed the predictive reliability of the model, with errors in the load and failure initiation within 2%. In addition, the crack propagation patterns observed in the experiments closely matched those predicted by the FEM. These results demonstrate the effectiveness and practical applicability of the proposed DNN-GRA framework for deriving performance-specific and experimentally validated lay-up configurations of CFRP automotive components.

## Figures and Tables

**Table 3:** Load and MoS according to punch stroke.

Lay-Up						Punch Stroke: 12 mm		Punch Stroke: 14 mm		Punch Stroke: 16 mm	
						Load (N)	MoS	Load (N)	MoS	Load (N)	MoS
0	60	30	0	60	0	1001.79	0.3024	1127.38	−0.8993	1406.14	−0.0909
0	30	30	40	75	0	990.23	0.2795	1114.38	0.0580	1388.32	−0.1039
15	0	75	45	0	45	1002.99	0.2230	1128.53	0.0092	1409.18	−0.1460
30	75	75	45	75	75	993.98	0.4222	1118.33	0.1563	1394.56	−0.0982
30	0	45	15	0	15	999.21	0.2794	1124.75	0.0704	1403.85	−0.0917
45	75	15	15	30	60	1021.02	0.3310	1146.93	0.0897	1432.99	−0.0982
45	45	30	30	45	60	1006.32	0.3192	1129.89	0.0908	1411.61	−0.0974
60	75	60	0	45	0	999.99	0.1462	1125.06	−0.0328	1405.09	−0.1714
75	60	15	60	45	15	1004.29	0.2235	1130.30	0.0291	1412.38	−0.1204
75	60	0	75	30	15	993.39	0.1967	1118.33	0.0071	1396.99	−0.1394

**Table 4:** Structure and parameter of DNN model.

Parameter	Value
Initial dataset	100% (2000)
Training dataset	70% (1400)
Test dataset	15% (300)
Validation dataset	15% (300)
Training data sampling method	Random sampling
Data normalization method	Min-Max normalization
Maximum iteration	300
Learning rate	0.001
Activation function	ReLU
Optimization algorithms	Adam
Hidden layer/Node	5/1000

**Table 5:** Prediction performance of trained DNN model.

Test Dataset Error		
MAE	RMSE	*R* ^2^
0.023	0.038	0.978

**Table 6:** Error comparison between FE-simulation and predicted value through DNN model.

	GRG_1_ (Balanced)		GRG_2_ (Load-Focused)		GRG_3_ (MoS-Focused)	
	Load (N)	MoS	Load (N)	MoS	Load (N)	MoS
FEM	1143.86	0.2159	1157.47	0.1179	1117.25	0.2401
DNN	1141.05	0.2253	1156.78	0.1173	1116.59	0.2477
Error (%)	0.25	4.35	0.06	0.51	0.06	3.17

**Table 7:** Error comparison between FE-simulation and experiment at punch stroke 14 mm.

	Case 1 (Balanced)		Case 2 (Load-Focused)		Case 3 (MoS-Focused)	
	Load (N)	Initial Fracture Stroke (mm)	Load (N)	Initial Fracture Stroke (mm)	Load (N)	Initial Fracture Stroke (mm)
FEM	1143.86	16.20	1157.47	15.20	1117.25	16.60
Experiment	1131.46	15.91	1164.83	15.05	1106.33	16.40
Error (%)	1.08	1.79	0.64	0.99	0.98	1.20

## Data Availability

The original contributions presented in this study are included in the article. Further inquiries can be directed to the corresponding author.

## Appendix A

**Table A1:** Pareto optimal set and results of GRG.

No.	Lay-Up						GRC_Load_	GRC_MoS_	GRG_1_	GRG_2_	GRG_3_
1	0	45	45	15	0	45	0.7338	0.8079	0.7709	0.7412	0.8005
2	0	45	45	30	0	45	0.7214	0.8297	0.7756	0.7323	0.8189
3	0	45	45	30	60	60	0.5888	0.8769	0.7329	0.6176	0.8481
4	0	45	45	45	60	60	0.5418	0.9482	0.7450	0.5824	0.9075
5	0	45	45	60	60	60	0.5139	0.9941	0.7540	0.5620	0.9461
6	0	45	60	0	15	45	0.7500	0.7837	0.7683	0.7560	0.7806
7	0	45	60	15	0	45	0.7296	0.8263	0.7780	0.7394	0.8167
8	0	45	60	15	15	45	0.7463	0.7978	0.7721	0.7515	0.7926
9	0	45	60	30	0	45	0.7187	0.8532	0.7860	0.7322	0.8397
10	0	45	60	30	60	60	0.5866	0.8946	0.7407	0.6175	0.8638
11	0	45	60	45	0	45	0.7014	0.8689	0.7852	0.7182	0.8521
12	0	45	60	45	0	60	0.6287	0.8756	0.7521	0.6534	0.8509
13	0	45	60	45	60	45	0.5847	0.9049	0.7448	0.6168	0.8729
14	0	45	60	45	60	60	0.5341	0.9774	0.7558	0.5785	0.9331
15	0	45	60	45	75	60	0.5481	0.9175	0.7328	0.5851	0.8806
16	0	45	60	60	60	45	0.5727	0.9171	0.7449	0.6072	0.8827
17	0	45	60	60	60	60	0.5000	1.0000	0.7500	0.5500	0.9500
18	15	45	60	0	15	45	0.8398	0.7016	0.7708	0.8261	0.7154
19	15	45	60	0	30	45	0.8106	0.7128	0.7617	0.8008	0.7226
20	15	45	60	0	45	30	0.8161	0.7031	0.7596	0.8048	0.7144
21	15	45	60	15	15	45	0.8014	0.7339	0.7677	0.7947	0.7407
22	15	45	75	15	15	45	0.8081	0.7216	0.7649	0.7995	0.7302
23	15	60	45	0	15	45	0.8090	0.7132	0.7612	0.7995	0.7228
24	15	60	45	0	60	30	0.7906	0.7499	0.7703	0.7866	0.7539
25	15	60	60	0	15	45	0.8053	0.7269	0.7661	0.7975	0.7348
26	15	60	60	0	60	30	0.7800	0.7564	0.7682	0.7777	0.7587
27	15	60	60	15	60	30	0.7703	0.7762	0.7733	0.7709	0.7756
28	15	60	60	30	75	30	0.7536	0.7810	0.7673	0.7564	0.7783
29	30	60	0	45	15	45	0.8634	0.6408	0.7521	0.8412	0.6631
30	30	60	0	60	15	45	0.8592	0.6471	0.7532	0.8380	0.6684
31	30	60	15	0	15	45	0.9262	0.5678	0.7470	0.8904	0.6037
32	30	60	15	0	30	45	0.9383	0.5626	0.7505	0.9008	0.6002
33	30	60	60	0	15	45	0.9208	0.5980	0.7594	0.8885	0.6303
34	30	60	60	0	30	45	0.9165	0.6010	0.7588	0.8850	0.6325
35	30	60	60	0	60	30	0.9232	0.5960	0.7597	0.8906	0.6288
36	30	60	75	0	60	30	0.9081	0.6232	0.7657	0.8797	0.6517
37	30	60	75	30	60	30	0.8725	0.6315	0.7520	0.8484	0.6556
38	45	30	0	0	30	45	0.9755	0.5128	0.7442	0.9293	0.5591
39	45	30	0	0	45	45	0.9641	0.5249	0.7445	0.9202	0.5688
40	45	30	0	15	60	30	0.9512	0.5253	0.7383	0.9086	0.5679
41	45	45	0	0	15	45	0.9772	0.5043	0.7408	0.9300	0.5516
42	45	45	0	0	30	45	1.0000	0.5000	0.7500	0.9500	0.5500
43	45	60	0	0	15	45	0.9417	0.5334	0.7376	0.9009	0.5742
